# Prevalence of Patellofemoral Pain Among Adults in Saudi Arabia

**DOI:** 10.7759/cureus.74818

**Published:** 2024-11-30

**Authors:** Wazzan Aljuhani, Lubna Aloufi, Mohammed E Alhafi, Abdulaziz A Alquraiqari, Reemah Alqahtani, Ayman Alamr, Muath S Alghamdi

**Affiliations:** 1 Department of Surgery, Ministry of National Guard Health Affairs, Riyadh, SAU; 2 Department of Surgery, King Abdullah International Medical Research Center, Riyadh, SAU; 3 Department of Surgery, King Saud Bin Abdulaziz University for Health Sciences, Riyadh, SAU; 4 Department of Surgery, Princess Nourah Bint Abdulrahman University, Riyadh, SAU; 5 Department of Surgery, Imam Mohammad Ibn Saud Islamic University, Riyadh, SAU; 6 Department of Surgery, Ministry of Health, Riyadh, SAU; 7 Department of Surgery, King Khalid University, Abha, SAU; 8 Department of Surgery, University of Bisha, Bisha, SAU; 9 Faculty of Medicine, King Abdulaziz University, Jeddah, SAU

**Keywords:** anterior knee pain questionnaire, knee pain, kujala anterior knee pain scale, patellofemoral pain, prevalence

## Abstract

Background and aim: Patellofemoral pain syndrome (PFPS) is a disease that clinically presents with retro-patellar and peripatellar pain that affects primarily physically active individuals. This study aims to estimate the prevalence of PFPS in Saudi Arabia and the variables that contribute to its occurrence.

Objectives: This multicenter, cross-sectional study aimed to explore the prevalence of patellofemoral pain in Saudi Arabia.

Methods: This study was conducted in Saudi Arabia. Self-structured questionnaires translated into Arabic were distributed among Saudi adults using Google Forms (Google, Mountain View, CA). The questionnaire comprised questions regarding socio-demographic characteristics, knee pain characteristics, and the Kujala Anterior Knee Pain Scale to evaluate the PFPS of the participants.

Results: Among the 278 Saudi adult participants, 53.6% were aged between 18 and 25 years and 52.9% were male. Additionally, 15.1% of patients were obese. Of the 24.5% of patients who experienced knee pain, 44.1% experienced right knee pain. The PFPS prevalence in this study was 9%. Factors associated with PFPS were unemployment and knee pain. However, no significant differences in PFPS in terms of age, sex, and body mass index (BMI) were observed.

Conclusions: The prevalence of PFPS in the Saudi population was low. PFPS was more prevalent among unemployed Saudis with knee pain. Age, sex, and BMI were not considered relevant to PFPS. Further investigations should be performed to determine the prevalence of PFPS in Saudi Arabia.

## Introduction

Young adult and teenage athletes who engage in sports involving leaping, cutting, and pivoting movements frequently experience retropatellar and peripatellar pain, which is clinically known as patellofemoral pain (PFP) [1]. One of the most prevalent diseases among physically active individuals aged between 15 and 30 years is the PFP syndrome (PFPS) [2]. PFP is usually induced by functional tasks, including ascending and descending stairs, crouching, and extended sitting. It is frequently accompanied by crepitus, clicking, catching, and a sense of giving way. Joint effusions are uncommon. Bilateral symptoms are often chronic and persist for years without improvement. Diagnosis can be established by focusing on the patient’s medical history and physical examination results. The incidence of PFPS among athletes in the United States is >25% [3]. However, little is known about the prevalence of PFP, particularly its risk factors among athletes in Saudi Arabia. Thus, this study aimed to evaluate the prevalence of PFP in Saudi Arabia and highlight its causes.

## Materials and methods

This retrospective cross-sectional study was conducted in Saudi Arabia. It aimed to assess the prevalence of PFP in the Saudi population. The study was based on a self-structured online questionnaire and the available literature with similar objectives. Our questionnaire used the Kujala Anterior Knee Pain Scale to assess anterior knee pain in the Saudi population [4]. The Kujala Anterior Knee Pain Scale is the standard test used in the field of orthopedic sports medicine to assess the prevalence of PFP. It is a self-reported test comprising 13 questions with a total possible score of 100. The scores reflect the symptoms and limitations of activity experienced by the individual; the lower the score, the greater the pain or disability. The questionnaires were distributed online using Google Forms (Google, Mountain View, CA). The questionnaire was translated into Arabic to suit the population, and one question was modified to include prostration, which is a common activity among Muslims. The Arabic Kujala score was translated and validated by research teams in Jordan [5]. All participants were informed that their data would remain anonymous and would be kept secure and accessible only by those with authorization. The survey was distributed online to the Saudi population. Data collection started in January 2022. Ethical approval was obtained from the Institutional Review Board of King Abdullah International Medical Research Center (approval number: IRB/2250/22; study number: NRC22R/447/09) on October 18, 2022). The procedures followed were in accordance with the ethical standards of the responsible committee on human experimentation (institutional or regional) and with the Helsinki Declaration of 1975, as revised in 2013. The coinvestigators reviewed the responses and entered them into a data collection sheet. Data were entered using Microsoft Excel (Microsoft Corporation, Redmond, WA) and exported to SPSS version 22 (IBM Corp., Armonk, NY) for analysis. Numerical data, such as age, are presented as the mean ± standard deviation. Categorical data, such as sex, are presented as frequencies and percentages. The main outcome of the participant is presented as the relative risk and 95% confidence interval. P < 0.05 was considered significant.

Questionnaire criteria

The prevalence of PFPS was assessed using the Anterior Knee Pain Questionnaire (AKPQ) [4], which includes 13 questions regarding the ability to perform several different activities and one question about pain. It is considered a valid and reliable tool that is easy to use [6,7]. The possible AKPQ scores range from 0 to 100 points. The cutoff score for the AKPQ was 83 points because scores of ≥83 points indicated no sign of anterior knee pain [8].

Statistical analysis

Descriptive statistics are presented as numbers and percentages. The relationship between PFPS and the socio-demographic characteristics of participants was evaluated using the chi-square test. The statistical level of significance was set as p < 0.05. All statistical data were calculated using SPSS.

Sample selection

**Figure 1:**
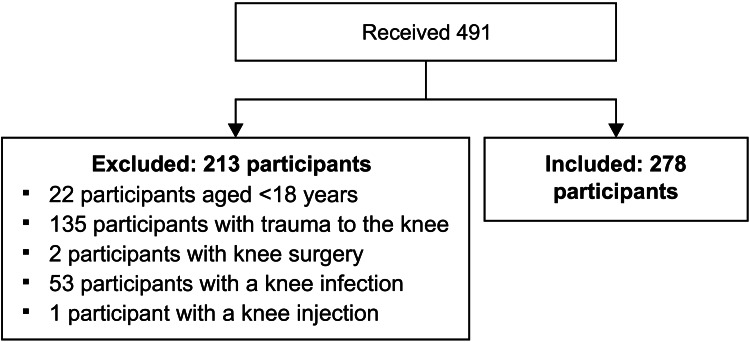
Sample selection.

Sample selection was performed, as shown in Figure 1.

## Results

We received completed questionnaires from 491 potential participants (response rate: 56.6%). Of these, 278 participants met the inclusion criteria. Table 1 presents the demographics and clinical characteristics of the participants. The most common age group was 18-25 years (53.6%), and more than half (52.9%) of the participants were male. Furthermore, 15.1% of the participants were obese (BMI ≥ 30 kg/m2). Nearly half (48.2%) lived in the central region and 41.7% were students. Additionally, 24.5% of participants experienced knee pain. Of these participants with knee pain, 44.1% experienced right knee pain.

**Table 1:** Participants’ demographics and clinical characteristics (n = 278).

Study variables	N (%)
Age group (years)	
18–25	149 (53.6%)
26–35	46 (16.5%)
>35	83 (29.9%)
Sex	
Male	147 (52.9%)
Female	131 (47.1%)
BMI (kg/m^2^)	
<18.5	27 (09.7%)
18.5–24.9	122 (43.9%)
25–29.9	87 (31.3%)
≥30	42 (15.1%)
Occupational status	
Employed	114 (41.0%)
Unemployed	48 (17.3%)
Student	116 (41.7%)
Knee pain	
No	210 (75.5%)
Yes	68 (24.5%)
Knee pain side (n = 68)	
Right	30 (44.1%)
Left	17 (25.0%)
Bilateral	21 (30.9%)

**Figure 2:**
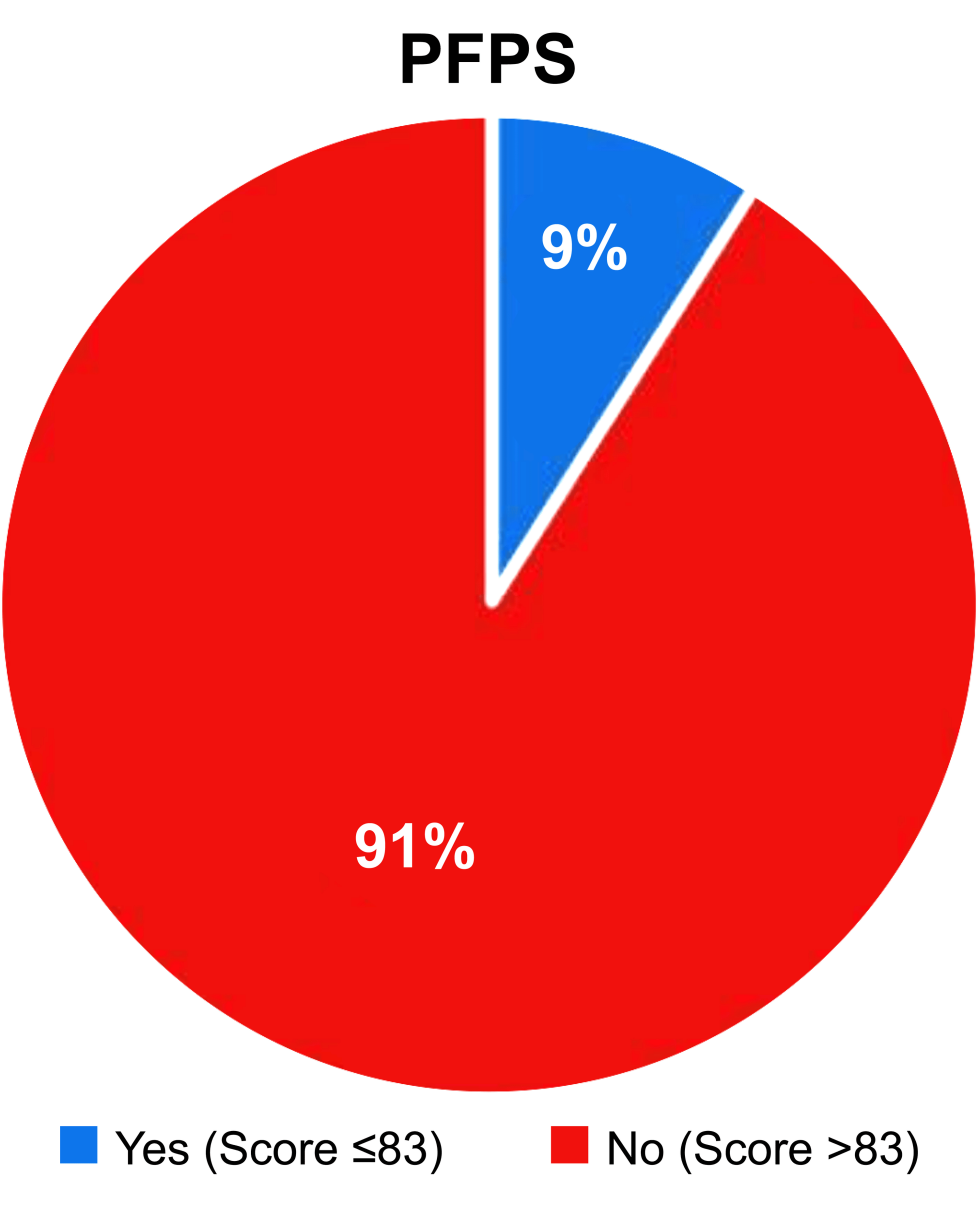
Prevalence of patellofemoral pain syndrome (PFPS) according to the Anterior Knee Pain Questionnaire (AKPQ) score.

As shown in Figure 2, according to the criteria of the AKPQ, the prevalence of PFPS was 9%. An evaluation of the relationship between PFP and participants’ socio-demographic characteristics (Table 2) showed that the prevalence of PFPS was significantly more common among unemployed participants (p = 0.034) and those experiencing knee pain (p < 0.001). In contrast, no significant associations were observed between the prevalence of PFPS and age (p = 0.867) and BMI (p = 0.064).

**Table 2:** Relationship between patellofemoral pain and socio-demographic characteristics of the participants (n = 278). ^*^ P-value was calculated using the chi-square test. ^†^ Significant at p < 0.05.

Characteristic	Patellofemoral pain		P-value^*^
	Yes, N (%) (n = 25)	No, N (%) (n = 253)	
Age			
≤25 years	13 (52.0%)	136 (53.8%)	0.867
>25 years	12 (48.0%)	117 (46.2%)	
Sex			
Male	14 (56.0%)	133 (52.6%)	0.743
Female	11 (44.0%)	120 (47.4%)	
BMI			
<25 kg/m^2^	9 (36.0%)	140 (55.3%)	0.064
≥25 kg/m^2^	16 (64.0%)	113 (44.7%)	
Occupational status			
Employed	8 (32.0%)	106 (41.9%)	0.034^†^
Unemployed	9 (36.0%)	39 (15.4%)	
Student	8 (32.0%)	108 (42.7%)	
Knee pain			
No	0	210 (83.0%)	<0.001^†^
Yes	25 (100%)	(17.0%)	

## Discussion

This study investigated the prevalence of PFPS among Saudi adults. Our results showed a low prevalence of PFPS (only 9%) in this population. This prevalence was lower than those observed in Riyadh [9] and Majmaah [10], which had a prevalence of 30.3% and 38.8%, respectively. Similarly, in Estonia [11], the prevalence of PFPS was 20.2%. Based on a systematic review and meta-analysis performed by Smith et al. [1], the annual prevalence of PFPS in the general population was 22.7% for adults and 28.9% for adolescents. However, among Pakistani students enrolled in sports science classes [12], 26.7%, 9.7%, and 63.5% of students had moderate, severe, and mild or no symptoms, respectively. Although PFPS was not highly prevalent in Saudi Arabia, it does not reflect those of other regions. Thus, clinicians should be aware of the early diagnosis and treatment of this condition.

Our study data indicated that PFPS was more prevalent among unemployed Saudis. However, the proportions of PFPS with age and BMI were not significantly different. These findings are comparable with those of a study performed in China [13] that reported no significant associations of PFPS with age and BMI. However, Mohammad and Elsais [9] observed a significant association between age and PFP; they reported an association between older age and increased risk of PFP. The burden of PFPS is physically and mentally detrimental to patients. According to the results of a systematic review by Maclachlan et al. [10], an individual with PFP might experience anxiety, depression, catastrophizing behaviors, and a fear of movement associated with pain, which could lead to reduced physical functions. This finding was supported by the results observed by Brady and Boonprakob [14], who found that chronic PFP greatly affected the psychological condition of students, especially those with high pain intensity, leading to less mindfulness, more catastrophizing behaviors, and more fear avoidance. Hence, psychological and physical treatments have been suggested for patients with chronic PFP.

Our study results found a higher PFPS prevalence in men (56%) than in women (44%), although the overall results did not reach statistical significance (p = 0.743). Similarly, a study performed in Thailand [14] found that the overall prevalence of chronic PFP involving knee pain was 43.3%, with a higher prevalence in men (48.2%) than in women (36.6%). However, this finding contradicts the findings of Mohammad and Elsais [9], who reported a higher PFP prevalence among women (72.3%) than among men (27.7%). Furthermore, the findings of Mohammad and Elsais concur with those of a study performed at the United States Naval Academy [15]. The prevalence of knee pain was 24.5% among our population, which is noteworthy. However, Aldharman et al. [8] reported a knee pain prevalence of 13.2%, with a higher prevalence among those younger than 40 years. Additionally, our results indicated that all participants who reported knee pain might have PFPS.

The limitation we faced during conducting this research was the online distribution of our survey limited its reach only to participants who were active online. Additionally, the data collected through self-reported surveys may be subject to recall bias.

## Conclusions

Our study found a 9% prevalence of PFPS in the Saudi population, which is lower than that reported in the literature. Furthermore, PFPS was more prevalent among unemployed Saudi individuals. However, no statistically significant differences in the prevalence of PFPS in terms of age, sex, and BMI were observed. Therefore, further longitudinal studies are necessary to establish a more comprehensive understanding of the prevalence of PFPS and its contributing factors in Saudi Arabia.
